# Commentary on: "Further studies are necessary in order to conclude a causal association between the consumption of monosodium L-glutamate (MSG) and the prevalence of the metabolic syndrome in the rural Thai population"

**DOI:** 10.1186/1743-7075-10-13

**Published:** 2013-01-22

**Authors:** Kate S Collison

**Affiliations:** 1 Diabetes Research Unit, Department of Cell Biology, King Faisal Specialist Hospital & Research Centre, P. O. Box. 3354, Riyadh, 11211, Saudi Arabia

## Abstract

See related article: http://www.nutritionandmetabolism.com/content/10/1/14

## 

Sir,

I read with considerable interest the epidemiological study by Insawang *et al*., which demonstrates an association between monosodium glutamate (MSG) intake and the prevalence of the Metabolic Syndrome in a rural Thai population [1]. It is important to point out that Insawang *et al*. did not claim that MSG causes the Metabolic Syndrome, they did however concluded that “elevated dietary MSG consumption is significantly associated with having the Metabolic Syndrome and being overweight in a Thai rural population”.

The present commentary by Dr Rogers [2] concerning the research by Insawang *et al*. stresses throughout that there is no supporting evidence for a direct causal relationship between MSG intake and the prevalence of Metabolic Syndrome and overweight. The relevance of this oft-repeated statement is questionable since Insawang *et al*. never proposed a direct causal relationship between MSG intake and the Metabolic Syndrome. Although the authors of this epidemiological study are under no obligation to provide evidence for a causal relationship, a number of issues were raised which make interesting points for discussion. One concern broached by Dr Rogers was that the authors failed to mention in their Discussion a previous publication regarding the Jiangsu Nutritional Study of 1227 Chinese adults [3], which did not show an association between MSG and obesity or weight gain over 5 years. In point of fact, the methodology and the conclusions drawn from that particular study were not only questioned, but seriously criticized [4]. Nevertheless, one of the reported findings was a significant association between MSG intake in 2002 and waist circumference five years later [4]. Although it is perhaps understandable that the authors Insawang *et al*. did not comment on the lack of association between MSG and body weight [3], they did discuss a separate publication concerning the exact same population of 1227 Chinese adults, from the same corresponding author, which showed a significant positive association between MSG intake and hypertension [5]. Elevated blood pressure is one of the five conditions which constitute the Metabolic Syndrome. The National Cholesterol Education Program Adult Treatment Panel III (NCEP) criteria require the presence of at least 3 of the following [6]:

[1] Hypertension, defined as elevated blood pressure defined as ≥ 130/85 mmHg.

[2] Abdominal obesity defined as waist circumference ≥ 102 cm or 40 inches (male), or ≥ 88 cm or 36 inches (female).

[3] Hyperglycemia, defined as elevated Fasting plasma glucose ≥ 110 mg/dl.

[4] Dyslipidemia, defined as elevated triglycerides ≥ 150mg/dL.

[5] Dyslipidemia, defined as presence of high-density lipoprotein cholesterol (HDL-C) ≤ 40 mg/dL (male), or ≤ 50 mg/dL (female).

Importantly, elevated body weight is not one of the criteria for the presence of the Metabolic Syndrome; and indeed, animal model systems indicate that MSG-obese rodents exhibit either lower body weights [7-10], or similar body weights compared to control animals [11,12], depending on the species and experimental conditions. However, impaired cardiovascular autonomic function, elevated arterial pressure, insulin resistance and dyslipidemia have all been documented in rodents exposed to MSG during the neonatal period at a time when the blood brain barrier is immature and vulnerable to excitotoxicity [13,14]. Moreover, neonatal exposure to non-physiological levels of MSG is a proven experimental methodology for inducing Metabolic Syndrome in rodents [15-18]; and sometimes referred to as “hypothalamic obesity” [19,20] due to the fact that high levels of glutamate may damage the hypothalamus and other areas of the brain which are rich in glutamate receptors [11,12]. Interestingly, increased hypothalamic inflammatory signaling and neuronal injury can also be induced in rodents consuming high fat diets [21-24]; and recent data also provides evidence of hypothalamic low-grade inflammation and gliosis in obese humans [24,25], which may impair the regulation of food intake and energy expenditure.

## Conclusion

[1] The authors of the epidemiological study associating MSG consumption with the prevalence of Metabolic Syndrome were under no obligation to provide a causal relationship between the two. [2] Under experimental conditions in rodents, non-physiological levels of MSG, or high levels of dietary fat may promote damage to the hypothalamus and other areas of the brain regulating energy expenditure. [3] In humans, obesity may be associated with hypothalamic damage. The commentary by Rogers has provided several interesting points of discussion.

## Competing interests

The author declares no competing interests, and has no industrial or personal disclosure.
